# Unconventional magnetization textures and domain-wall pinning in Sm–Co magnets

**DOI:** 10.1038/s41598-020-78010-0

**Published:** 2020-12-03

**Authors:** Leonardo Pierobon, András Kovács, Robin E. Schäublin, Stephan S. A. Gerstl, Jan Caron, Urs V. Wyss, Rafal E. Dunin-Borkowski, Jörg F. Löffler, Michalis Charilaou

**Affiliations:** 1 Laboratory of Metal Physics and Technology, Department of Materials, ETH Zurich, 8093 Zurich, Switzerland; 2 Ernst Ruska-Centre for Microscopy and Spectroscopy with Electrons, and Peter Grünberg Institute, Forschungszentrum Jülich, 52425 Jülich, Germany; 3 Scientific Center for Optical and Electron Microscopy, ETH Zurich, 8093 Zurich, Switzerland; 4 Arnold Magnetic Technologies, 5242 Birr-Lupfig, Switzerland; 5 Department of Physics, University of Louisiana at Lafayette, Lafayette, LA 70504 USA

**Keywords:** Magnetic properties and materials, Magnetic properties and materials

## Abstract

Some of the best-performing high-temperature magnets are Sm–Co-based alloys with a microstructure that comprises an $\hbox {Sm}_2\hbox {Co}_{17}$ matrix and magnetically hard $\hbox {SmCo}_5$ cell walls. This generates a dense domain-wall-pinning network that endows the material with remarkable magnetic hardness. A precise understanding of the coupling between magnetism and microstructure is essential for enhancing the performance of Sm–Co magnets, but experiments and theory have not yet converged to a unified model. Here, transmission electron microscopy, atom probe tomography, and nanometer-resolution off-axis electron holography have been combined with micromagnetic simulations to reveal that the magnetization state in Sm–Co magnets results from curling instabilities and domain-wall pinning effects at the intersections of phases with different magnetic hardness. Additionally, this study has found that topologically non-trivial magnetic domains separated by a complex network of domain walls play a key role in the magnetic state by acting as nucleation sites for magnetization reversal. These findings reveal previously hidden aspects of magnetism in Sm–Co magnets and, by identifying weak points in the microstructure, provide guidelines for improving these high-performance magnetic materials.

## Introduction

Sm–Co-based materials are some of the best-performing permanent magnets available today, particularly for vital high-temperature and precision applications thanks to their high Curie temperatures and large magnetocrystalline anisotropy^1–3^. Extensive research and industrial development in the past few decades have led to a significant improvement in their magnetic performance^4^. One such example is a highly engineered Sm–Co-based system that consists of a cellular microstructure with a $\hbox {Sm}_2\hbox {Co}_{{17}}$ matrix enclosed by $\hbox {SmCo}_5$ cell walls and intersected by the so-called Z phase (Zr-rich platelets) perpendicular to the *c*-axis and magnetic easy axis of the $\hbox {Sm}_2\hbox {Co}_{{17}}$ matrix^1,5,6^. This characteristic geometry results from tailored aging-heat treatments^1^, and corresponds to a network of magnetically intertwined phases of different magnetic hardness.

Magnetic properties due to combinations of these structures are highly tunable because they make use of the high saturation magnetization of the $\hbox {Sm}_2\hbox {Co}_{{17}}$ matrix and the strong magnetocrystalline anisotropy of the $\hbox {SmCo}_5$ cell walls^7,8^. Particular attention has been devoted to modeling the cellular microstructure in order to predict the coercivity^9–12^. The coercivity enhancement in these cellular Sm–Co magnets emerges from the difference between the magnetocrystalline anisotropy of the two phases and consequently the difference in domain-wall energy^9^. Conventional wisdom states that this microstructure constitutes a pinning system for domain walls, but the exact magnetization processes remain elusive despite the intense activities that have been performed to understand the interaction of domain walls with the $\hbox {SmCo}_5$ cell walls^13–19^.

Magnetic imaging experiments, by means of Lorentz transmission electron microscopy (LTEM), magnetic force microscopy, and Kerr microscopy, have revealed that domain walls follow the $\hbox {SmCo}_5$ cell-wall morphology^17,19–24^, thus confirming strong pinning at the cell-wall boundaries. Theory and experiment, however, have yet to converge on the role of the Z phase in determining the magnetic properties of this material^9–12^. Forward modeling of the Z phase is impeded by the fact that the material parameters of this phase cannot be easily estimated, because the thickness of the Z phase can be as thin as 1–2 atomic layers (the thickness varies from material to material), and thus cannot be compared with measurements on bulk samples^25^. Hence, high-resolution imaging of the magnetization textures is crucial to elucidate the interplay between the matrix, the cell walls, and the Z phase, and unveil the magnetization processes that are at play in the cellular Sm–Co magnets.

Here we present a detailed study of the magnetic state in a cellular Sm–Co magnet containing Fe, Cu and Zr, where we correlate atomic-resolution TEM and atom probe tomography (APT) with high-resolution LTEM and off-axis electron-holography (EH) imaging of the domain-wall structure. We systematically compare the experimental results with detailed micromagnetic simulations. By matching experiments and theory one-to-one we show that the nanoscale magnetization processes in cellular Sm–Co magnets stem from an interplay between pinning at the $\hbox {SmCo}_{{5}}$ cell-wall boundaries and curling instabilities at the intersections of all three phases.

**Figure 1:**
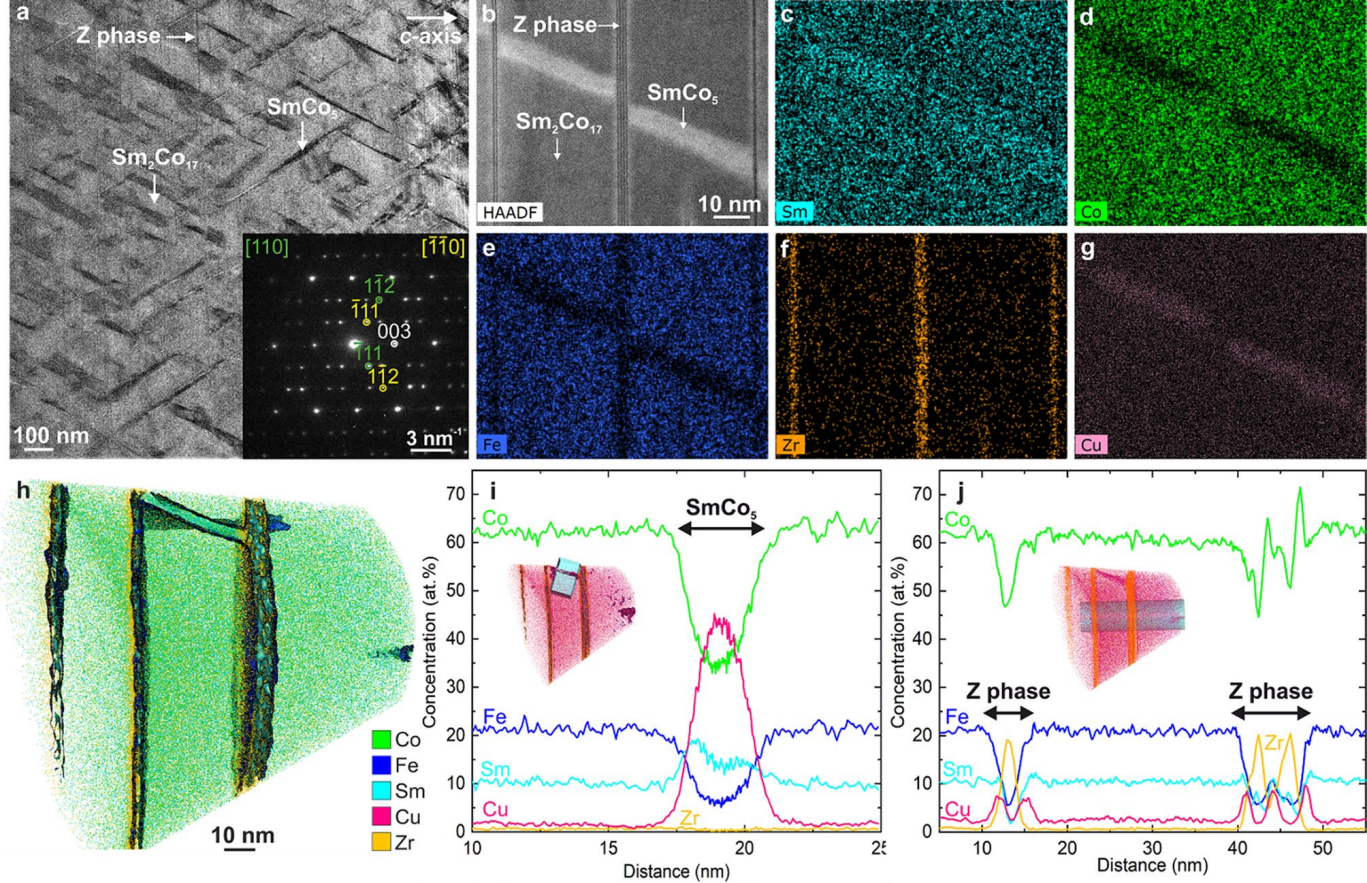
Sm–Co microstructure. (**a**) Bright-field TEM image of a Sm–Co magnet showing the $\hbox {Sm}_2\hbox {Co}_{{17}}$ matrix (light grey) enclosed by $\hbox {SmCo}_5$ cell walls (dark grey), with the entire structure intersected by the Z phase. The corresponding diffraction pattern, shown in the bottom right corner, reveals twinning of the [110] (green) and [$\bar{1}\bar{1}$0] (yellow) directions (see Supplementary Figure 1). (**b**) High-angle annular dark-field (HAADF) scanning TEM image showing the details of the microstructure, accompanied by EDX chemical maps of (**c**) Sm, (**d**) Co, (**e**) Fe, (**f**) Zr and (**g**) Cu. (**h**) Atomic-resolution APT reconstruction with the isoconcentration surfaces of Zr and Fe, exhibiting flat Z-phase platelets (vertical) and a twisted $\hbox {SmCo}_5$ cell wall (in the top middle between two flat Z-phase platelets). Concentration plots, as indicated by insets, of individual elements show that (**i**) Cu accumulates in the middle of the cell wall, while Sm increases non-symmetrically across the cell wall, and (**j**) Zr peaks in the middle of the Z phase, while Cu accumulates at the interface between the Z phase and the $\hbox {Sm}_2\hbox {Co}_{{17}}$ matrix.

**Figure 2:**
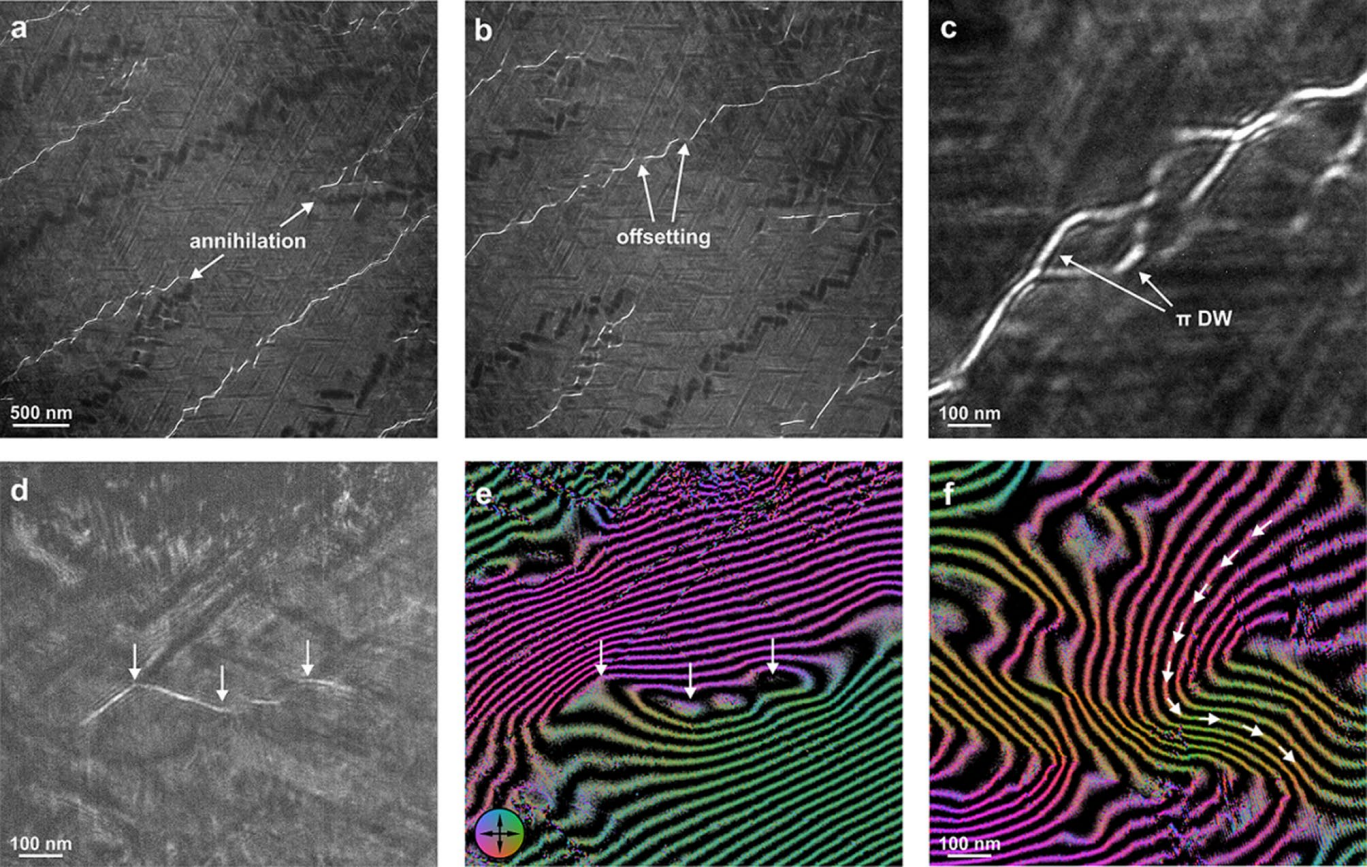
Magnetic structures in a Sm–Co magnet. DW structures imaged by LTEM in 0.5 mm (**a**) over- and (**b**) underfocus are characterized by DW annihilation and offsetting, *i*.*e*., apparent discontinuities in the DWs. (**c**) Higher magnification reveals a topologically non-trivial structure with branching and alternating $\pi$ and 2$\pi$ magnetization profiles. A comparison of (**d**) a Fresnel defocus image and (**e**) a magnetic induction map extracted from off-axis EH of the same region (arrows indicate the same location) reveals a $\pi$ DW involving vortex-like curling. (**f**) Magnetic induction map of a complex magnetic state consisting of DWs with a wide range of angles (the arrows follow the magnetic induction direction). The phase difference between adjacent contours in the induction maps corresponds to 2$\pi$ radians, and the different contour spacing in panels e and f results from a different specimen thickness.

## Results

The Sm–Co sample in our study has an overall chemical composition of Sm(Co,Fe,Cu,Zr)$_{7.57}$ with minor amounts of oxygen (see the Methods section). Figure 1 shows for this sample an overview of its microstructure, revealing the typical $\hbox {Sm}_2\hbox {Co}_{{17}}$ matrix enclosed by $\hbox {SmCo}_5$ cell walls and intersected by the Z-phase platelets. A close-up view in Fig. 1b and corresponding energy-dispersive X-ray spectroscopy (EDX) chemical maps in Fig. 1c–g show interfaces between the three phases and confirm that the Zphase is rich in Zr. The thickness of the interface between the $\hbox {Sm}_2\hbox {Co}_{{17}}$ matrix and the $\hbox {SmCo}_5$ cell walls ranges from atomically sharp to 2 nm (visible as blurry contrast), whereas the interfaces with the Z phase are always atomically sharp. The *c*-axis of the crystal structure (see Fig. 1 in the Supplementary Material) lies inside the TEM lamella, and the Z platelets are always perpendicular to the *c*-axis^6^.

The APT reconstruction in Fig. 1h shows the isoconcentration surfaces of Zr and Fe with concentration values of 9.8 and 13.5 at%, respectively, and reveals four perfectly flat Z platelets, where the two rightmost are actually so close that they are visible only as one wide platelet. In the top middle part of Fig. 1h, a twisted $\hbox {SmCo}_5$ cell wall can be seen between two Z platelets. The twisted shape explains why in Fig. 1a different interfaces between the matrix and the cell walls have different sharpness. The concentration profiles of individual elements across a $\hbox {SmCo}_5$ cell wall and a Z platelet are shown in Fig. 1i,j, respectively (across the blue areas in the inset figures). While the overall Cu concentration in the sample is less than 10 at%, it peaks in the cell walls at 43 at% with a Gaussian-like distribution, which is expected to critically affect the magnetic properties^24^. The increase in Sm across the $\hbox {SmCo}_5$ cell wall appears to be non-symmetric, which may be due to applying a one-dimensional concentration profile to a twisted cell wall. As expected, Zr increases across the Z platelets, but, surprisingly, Cu segregates at the interfaces between the platelets and the matrix, which has a significant impact on the magnetic performance. Therefore, further nanoscale segregation and clustering studies are encouraged. The $\hbox {SmCo}_5$ cell walls are typically around 200 nm wide across their widest region and approximately 15 nm thick (Fig. 1a), and the Z platelets are at most 5 nm thick (Fig. 1b) and thus consist of only a few atomic layers (some platelets consist of only 1–2 atomic lattice planes). As we will discuss below, it is the thickness of the Z platelets that crucially determines the magnetic properties.

Figure 2 shows magnetic imaging studies of the cellular Sm–Co magnet in a thermally demagnetized (magnetically pristine) state. Figure 2a,b are over- and underfocus Fresnel-mode LTEM images of the same area. Domain walls (DWs) appear as alternating sharp and bright contrast (convergent), or blurry and dark contrast (divergent), depending on the sign of the defocus and the direction of the magnetization profile along the DW. Changing the sign of the defocus inverts the contrast, *i*.*e*. the same DWs have opposite contrast in over- and underfocus images. These LTEM images reveal a complex DW pattern that follows exactly the microstructure of the material, in agreement with what is already documented in the literature^17,19–21^. We also observe apparent discontinuities in the DWs, where they seem to abruptly stop and shift approximately 50 nm sideways from their direction. We call these discontinuities DW offsetting, and two examples of them are marked in Fig. 2b.

Even though LTEM does not enable a quantification of the magnetization direction along the DW profile, considering the high uniaxial anisotropy in the system we infer that these are Bloch-type $\pi$ DWs^17^. From the intensity profiles of divergent DWs at different defocus values (see Supplementary Figure 2), we measured the full width at half-maximum (FWHM) of the DWs, which is $4\pm 2$ nm. This corresponds to the DW-width parameter $\delta _ {\rm DW} = \sqrt{A/K_ {\rm u}}$, where *A* is the exchange stiffness and $K_ {\rm u}$ is the uniaxial magnetocrystalline anisotropy^26^. The theoretical value of $\delta _ {\rm DW}$ is 2.7 nm for $\hbox {Sm}_2\hbox {Co}_{{17}}$ and 1.2 nm for $\hbox {SmCo}_{{5}}$ (for the values of *A* and $K_ {\rm u}$ used, see Methods section). Our experimental result suggests that the DWs are mostly located in the magnetically less hard $\hbox {Sm}_2\hbox {Co}_{{17}}$ phase, reminiscent of an exchange spring magnet^27,28^. The DW-width parameter should be distinguished from the DW width $\pi \sqrt{A/K_ {\rm u}}$^29^, which describes the width of the full rotation from 0 to $\pi$, and thus does not correspond to what is measured by LTEM.

While the zig-zag DW structure is well known^30^, here we observe new and rather unexpected DW patterns at some of the intersections of the three phases, where DWs of opposite sense meet and annihilate each other, leaving a trivial ferromagnetic state (marked with arrows in Fig. 2a,b). Surprisingly, topologically complex structures bounded by two $\pi$ DWs of the same sense, *i*.*e*. with a total winding of 2$\pi$, can also be observed, as shown in Fig. 2c. The unwinding of such regions is non-trivial and requires a violation of topological constraints^31^.

We have complemented LTEM with off-axis EH to gain in-depth information on the direction of the local magnetic field inside the sample. Figure 2d shows an LTEM image of a DW pinned to a $\hbox {SmCo}_5$ cell wall with contrast that varies in intensity. The DWs may be tilted inside the sample and therefore overlap with adjacent magnetic domains, which might result in such contrast. However, a magnetic induction map extracted from off-axis EH of the same area, shown in Fig. 2e, provides more information about this magnetic structure. The DW has a winding of $\pi$ and the magnetic field curls around the $\hbox {SmCo}_5$ cell wall, forming closed loops reminiscent of magnetic vortices (indicated by the middle arrow). This curling may explain the variation of contrast intensity in LTEM images. Interestingly, Fig. 2e reveals another DW in the top left corner, which is not visible in LTEM in Fig. 2d. Figure 2f shows that the magnetic texture in some areas can be so complex that some DWs do not have a well-defined angle; instead their angle varies between $\pi /6$ and $\pi /2$. These exotic magnetization textures are closely correlated with the microstructure and indicate that topological aspects need to be considered in order to correctly interpret the magnetic state.

**Figure 3:**
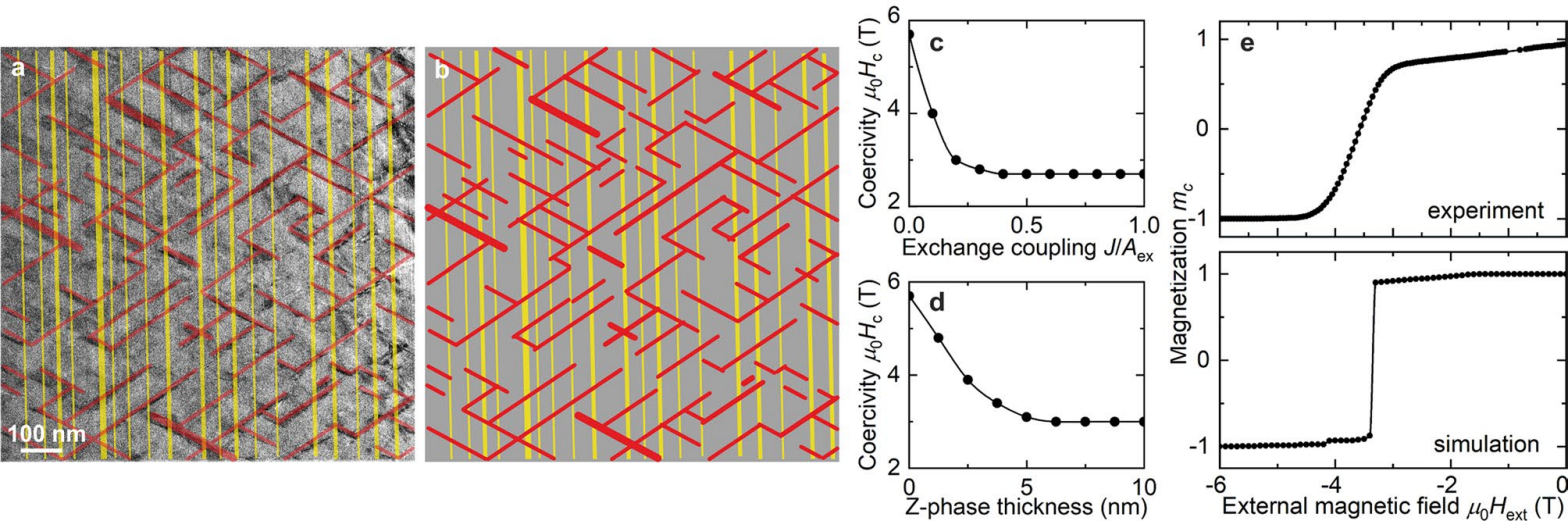
Modelling the microstructure of Sm–Co magnets. (**a**) Modelling of the microstructural features in Sm–Co, as seen in Fig. 1a, to create (**b**) a model with the $\hbox {Sm}_2\hbox {Co}_{{17}}$ matrix (grey), the $\hbox {SmCo}_5$ cell walls (red), and the Z phase (yellow). (**c**) Simulated dependence of the coercivity as a function of exchange coupling (the same at all interfaces), illustrating that smaller exchange between the phases leads to a higher coercivity. Using these results, we can compare the theoretical coercivity with that of real samples. (**d**) Simulated dependence of the coercivity on the thickness of the Z phase, showing that it increases significantly with decreasing thickness. (**e**) Comparison between an experimentally measured $m_c(H)$ demagnetization curve at *T* = 300 K and a simulated loop along the easy axis. By matching the simulation to the 300 K experimental data, we obtained the values of the exchange stiffness in the system, which are listed in the Methods section. The external field is applied parallel to the *c* axis, *i*.*e*., perpendicular to the Z-phase platelets.

Given the elaborate microstructure, these observations raise various questions regarding the magnetic state in cellular Sm–Co magnets. In order to obtain further insight, we performed detailed high-resolution micromagnetic simulations to elucidate the formation of the observed complex domain patterns. To this end, it is imperative that we fully consider the real microstructure, and thus we constructed a simulation system directly from the TEM images shown above. Figure 3a,b shows how we truncated the microstructure in order to model the system with the three phases having the same geometry and scale.

In our simulations, we considered the ferromagnetic exchange and uniaxial anisotropy energies, the dipole-dipole interactions, and the exchange energy between the three different phases. The material parameters (*A*, $K_ {\rm u}$, and saturation magnetization $M_ {\rm s}$) are well known and were taken from the literature (see Methods)^6,24,25,32^. The exchange-interaction energy between the phases is unknown. We therefore performed parametric micromagnetic studies where we varied the exchange interaction and compared the theoretical hysteresis curve with the experimental data, thus deducing the correct values by matching simulations to experiments (Fig. 3c–e). These values are listed in the Methods section.

As previously mentioned, the precise material properties of the Z phase are unknown because the exact chemical composition is unclear and the platelets can be as thin as a single-atomic layer (see Fig. 1b). All material parameters, e.g., exchange stiffness, saturation magnetization, and magnetocrystalline anisotropy, are affected by the reduced dimensions of the platelets and their interfaces^33^. It is known from thin-film studies that the anisotropy is the most sensitive property and changes drastically depending on the thickness and local atomic arrangements^34^. Given the small volume of the Z phase, its internal magnetic field is strongly affected by the strong exchange field of the surrounding Sm–Co. We therefore simulated two scenarios, in which we assumed that: (1) the Z-phase platelets have their bulk value of $K_ {\rm u}$^6,25^, or (2) the anisotropy of the Z phase is significantly smaller than that of the other two phases. From a comparison of the experimental and theoretical *M*(*H*) curves, we found that the simulations matched the experimental observations only if $K_ {\rm u}$(Z phase) $<< K_ {\rm u}$($\hbox {Sm}_2\hbox {Co}_{{17}}$ or $\hbox {SmCo}_5$). Hence, in the rest of the discussion, we assume that $K_ {\rm u} \approx 0$ for the Z phase.

Furthermore, in order to make our simulation quantitatively comparable to the experimental results, we matched the theoretically predicted coercivity with the experimentally measured coercivity by varying the exchange energy between the three phases. To perform the calibration, we used at least 10 different values for the exchange coupling of each interface. It was possible to eliminate some of them in the beginning, but in total we performed more than 250 simulations. For the sake of brevity, a simplified case, where the exchange coupling at all interfaces is the same, is shown in Fig. 3c. It demonstrates our general finding that the coercivity increases with decreasing exchange energy. This supports experiments that show that increasing Cu content leads to a higher coercivity, depending on the compositional gradient at the boundary^22,24^. This is due to the formation of Cu-rich interfaces between the magnetic phases, which decrease the exchange coupling between the $\hbox {Sm}_2\hbox {Co}_{{17}}$ matrix and the $\hbox {SmCo}_5$ cell walls. The effect of Cu on reducing *A*, $K_ {\rm u}$, and $M_ {\rm s}$ for the cell walls is further discussed in the context of Supplementary Figure 3.

The simulations illustrate that changing the thickness of the $\hbox {SmCo}_5$ cell walls does not modify the magnetic performance strongly, in agreement with experiments^35^ and theory^36^, showing that the pinning field is saturated for a $\hbox {SmCo}_5$ thickness of more than 4 nm. This contradicts previous predictions stating that the $\hbox {SmCo}_5$ thickness should be at least three times the exchange length ($3\delta _ {\rm exc} \approx 20$ nm) for effective DW pinning^10,12^. The pinning, however, is a complex process and depends strongly on the Cu content in the $\hbox {SmCo}_5$ cell walls^37^. In our experiments we have found a Cu-composition gradient, but because the variation of the magnetic material parameters as a function of Cu is unknown, we modeled $\hbox {SmCo}_5$ with a homogeneous Cu enrichment and took into account the effect of a Cu-composition gradient by varying the exchange coupling at the cell-wall boundaries. Here we found that the coercivity strongly depends on the Z-phase thickness (see Fig. 3d). In the absence of the Z phase, the coercivity has a maximum value of 5.7 T, while it decreases significantly with increasing Z-phase thickness up to 5 nm, where it reaches a minimum of 3 T and then remains constant. Although the Z phase cannot be completely eliminated because it is considered to act as a diffusion pathway during the formation of the $\hbox {SmCo}_5$ cell walls^20^, these results explain recent experimental observations, where the magnetic performance deteriorated with increasing Z-phase thickness^19^. As we will discuss below, smaller thickness impedes magnetization curling and hence a stronger external field is required to initiate the magnetization reversal process, which begins at the intersections of the Z phase and $\hbox {Sm}_2\hbox {Co}_{{17}}$ cells.

In the simulations that we discuss in the following paragraph, the thicknesses of the cell boundary and the Z phase were derived directly from the TEM images, where we have 10 nm thick $\hbox {SmCo}_5$ cell walls and 1 to 5 nm thick Z-phase platelets. Figure 3e shows an experimentally measured (see Methods) and a simulated $m_c(H)$ demagnetization curve ($m_c = M/M_{\text {s}}$) along the easy axis, confirming the agreement between experiment and theory, specifically the value of the remanence $M_ {\rm r} = 0.95 M_ {\rm s}$ and a gradual decrease of the magnetization prior to the full magnetization switching at − 3.4 T. Our simulations indicate that the gradual decrease is due to the Z phase switching earlier than the rest of the material, which is yet another confirmation that the Z phase does not exhibit significant magnetocrystalline anisotropy compared to the other two phases. Note that the $m_c(H)$ curves are not identical, because the experiment was performed on a bulk sample, where we have gradual switching of parts of the material, while the simulations consider a single thin lamella. Furthermore, the lamella with a thickness/width aspect ratio of about 1/1000 has an additional shape anisotropy, though much smaller than the magnetocrystalline anisotropy $K_ {\rm u}>>\mu _0 M_ {\rm s}^2/2$. Nevertheless, here we demonstrate that our work bridges length scales because we compare our nanometer-resolution model to macroscopic measurements of the magnetization and adjust the details of the model on the nanoscale to match it with the bulk behavior.

**Figure 4:**
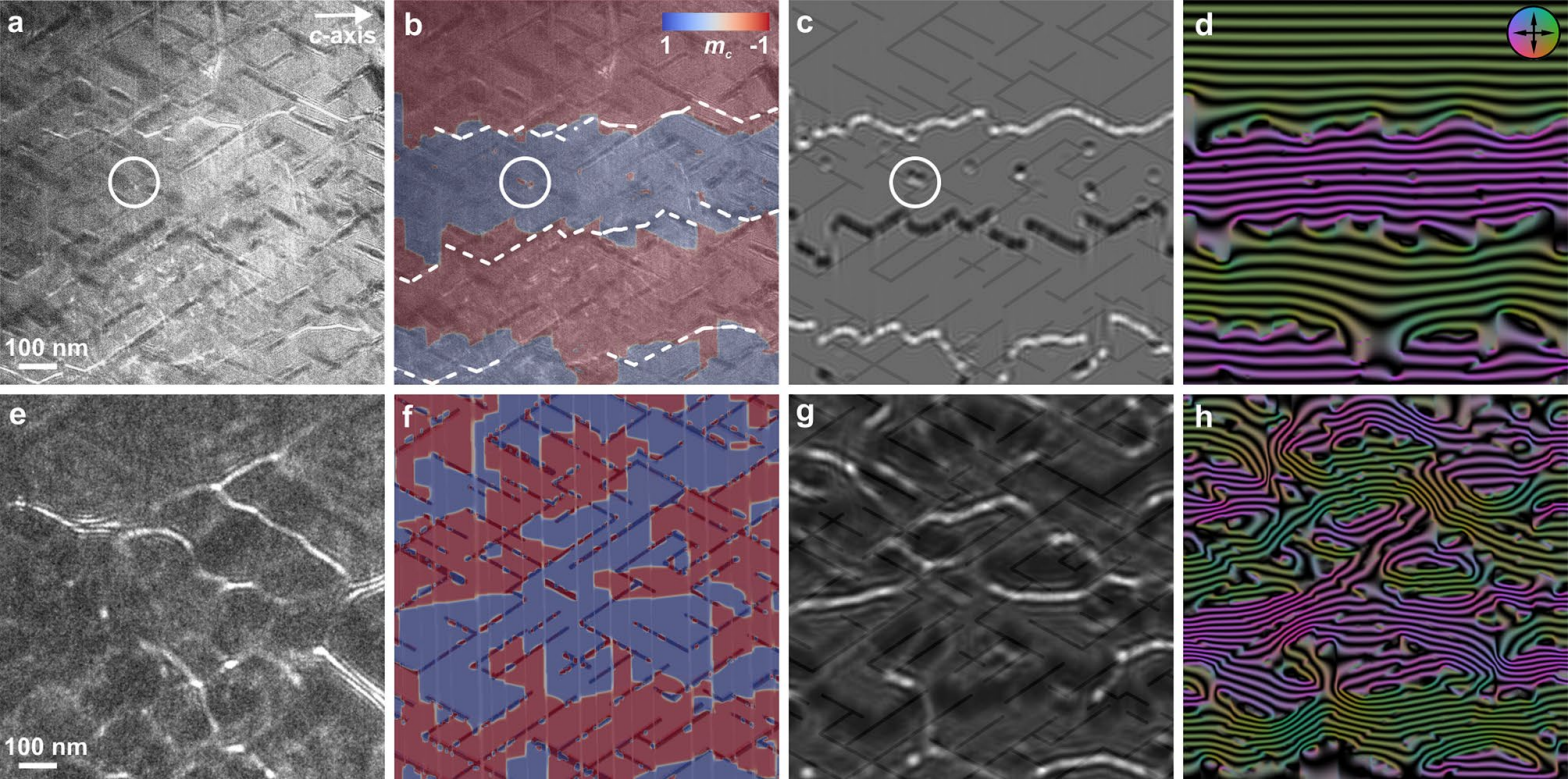
Comparison between experimental observations and theoretical predictions of the magnetization in Sm–Co. (**a**) LTEM image at 0.24 mm overfocus of the region shown in Fig. 1a, revealing four magnetic domains separated by DWs. (**b**) Micromagnetic simulation of the magnetization from the same microstructure superimposed on panel a. (**c**) A Fresnel defocus image and (**d**) a magnetic induction map simulated based on panel b. The images show a distinct resemblance of the DW network in theory and experiment, namely the pinning at $\hbox {SmCo}_5$ cell walls and the offsetting by the Z phase; the white circles indicate one of the small domains with opposite magnetization. (**e**) Fresnel defocus image at 0.8 mm overfocus of the remanent state. The region is not the same as in panel a, because it could not be found after saturating the lamella. (**f**) Simulation of the remanent state of panel a (used instead of panel e for a direct comparison with the thermally demagnetized state). Consecutive domains of opposite magnetization are present at the $\hbox {SmCo}_5$ cell walls. The corresponding (**g**) Fresnel defocus image and (**h**) magnetic induction map of the remanent state once again illustrate very good agreement between experiment and theory, and specify in particular the branching DW network pinned at the $\hbox {SmCo}_5$ cell walls. The phase difference between adjacent contours in the induction maps is 2$\pi$.

**Figure 5:**
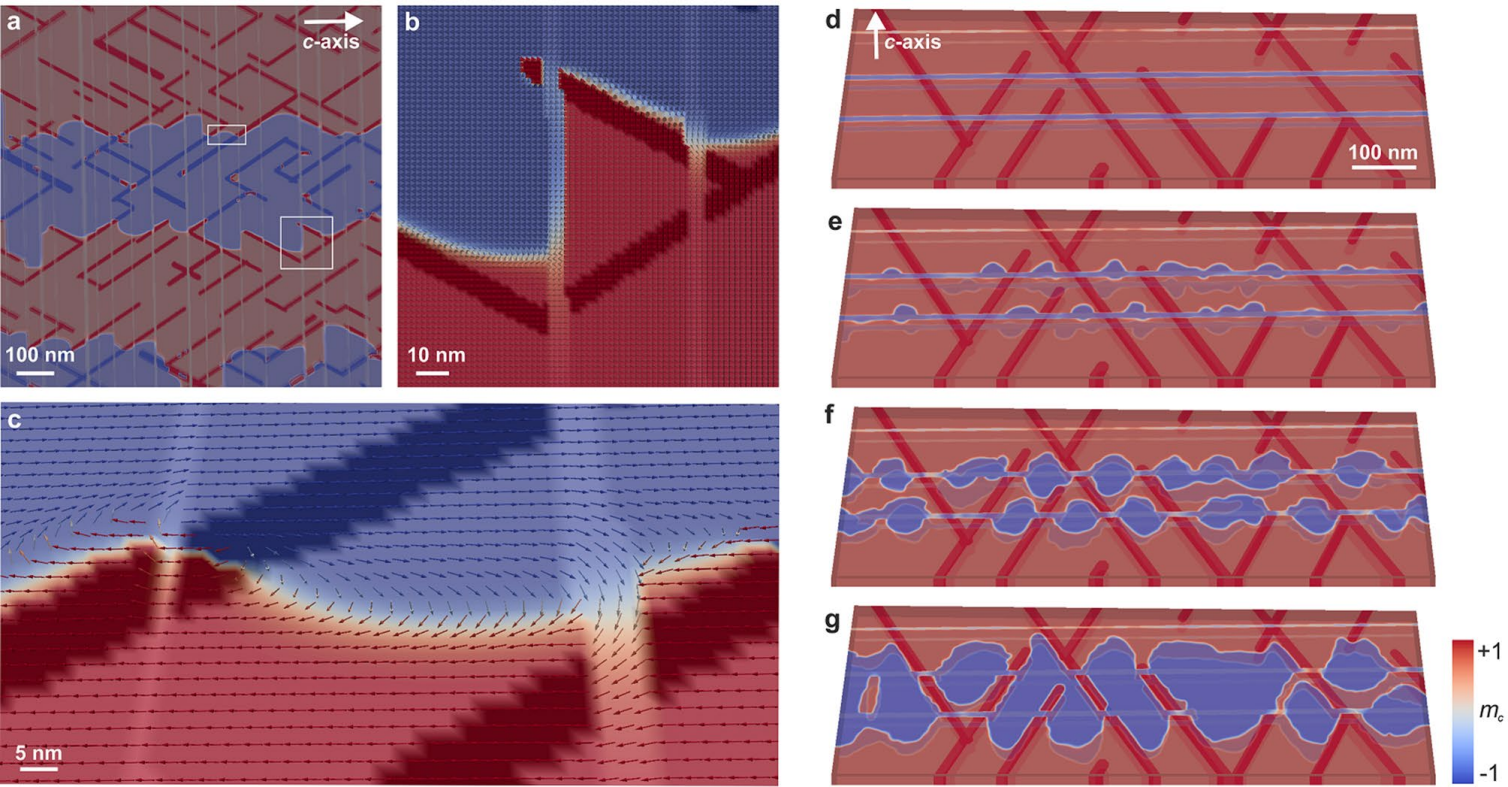
Simulations of 3D DW structures and their nucleation in Sm–Co magnets. (**a**) Structure with four domains in a demagnetized state showing that the DWs are pinned by the microstructural features, specifically the $\hbox {SmCo}_5$ cell walls, and are offset by the Z phase. (**b**) Close-up of the area marked with a square in panel a, revealing that the DWs are mostly situated inside $\hbox {Sm}_2\hbox {Co}_{{17}}$ and illustrating in detail how DW offsetting occurs at the Z phase. (**c**) Close-up of the area marked with a rectangle in panel a, illustrating the magnetization texture at an intersection of all three phases, where a DW is injected into the hard phase through a Z-phase platelet, consequently changing the DW width. (**d**–**g**) Time evolution of the magnetization reversal at a slightly supercritical field, *i*.*e*., larger than the switching field, parallel to the *c*-axis: as time progresses domains with magnetization parallel to the field start nucleating at the Z phase and spread into the $\hbox {Sm}_2\hbox {Co}_{{17}}$ matrix, but their growth is impeded by the $\hbox {SmCo}_5$ cell walls. The time step between each figure is 0.1 ns.

In order to study the magnetization texture in cellular Sm–Co magnets, we compare in the following simulated DW structures with those observed in the experiment. Figure 4a shows an experimental Fresnel defocus image at 0.24 mm overfocus of the region shown in Fig. 1a in the thermally demagnetized state, which contains magnetic domains separated by three DWs with a characteristic zig-zag shape following the microstructure. In our simulations, we initialized the system with three straight DWs and ran it for 1 ns to allow them to relax into the equilibrium state. The resulting magnetization texture is overlaid in Fig. 4b onto its corresponding TEM image. The positions of the DWs in the experimental LTEM image (white intersected lines) and the simulated magnetization image match very closely. In fact, in both cases the DWs follow precisely the microstructure. Additionally, small magnetic domains with opposite magnetization approximately 5 nm wide are present (white circles in Fig. 4), which form because of a strong pinning to $\hbox {SmCo}_5$. In order to directly correlate the micromagnetics with the experimental observations, a magnetic phase shift image has been calculated based on the micromagnetic results (see Methods). From this a Fresnel image and a magnetic induction map were reconstructed and are shown in Fig. 4c,d, respectively. A close match between theory and experiment is apparent, as the simulated images contain features, such as DW offsetting and curling, identical to those observed in Fig. 2. Additionally, the micromagnetic simulation reveals that the curling is out-of-plane (shown later in Fig. 5).

Furthermore, we ramped up the magnetic field to saturate the sample in both the experiment and the simulations and then removed the field to observe the remanent state. As we know from Fig. 3e, this corresponds to $M = 0.95 M_ {\rm s}$, meaning that one would expect the magnetization to be nearly uniform in the remanent state, but surprisingly this is not the case. Figure 4e, which shows an experimental Fresnel image of the remanent state (after applying an external field of 6 T), reveals a state with a complex network of domains separated by branching DWs (note that the region shown in Fig. 4e is not the same as in Fig. 4a.). This state represents initial nucleation stages of the magnetization process. Some DWs have strong contrast with multiple lines of three or more satellites, which are only visible for $\pi$ DWs that are perfectly edge-on (perpendicular to the lamella surface). Non-perpendicular DWs usually form weak and fading satellites. In Fig. 4f, the simulated magnetization state, again closely matching the experiment, contains a large number of small domains with opposite magnetization pinned to the $\hbox {SmCo}_5$ cell walls. These are the smallest possible domains, around 5 nm wide, and are constrained by the DW width. We again computed a magnetic-phase image from the micromagnetic simulation, from which we extracted a Fresnel defocus image and a magnetic induction map, shown in Fig. 4g,h, respectively. The magnetic texture shows a good match with the experiment, particularly with respect to the complex DW network, and the magnetic induction map reveals vortex-like out-of-plane curling in the remanent state.

Having confirmed the validity of our simulations, we took a deeper look into them beyond the experimental limitations. Figure 5 shows the magnetization as contour plots overlaid onto the microstructure. Note the prominent resemblance of Fig. 5a with the Fresnel defocus images of Fig. 2, *i*.*e*. the DWs follow exactly the $\hbox {SmCo}_5$ cell-wall geometry, including the offsetting by the Z-phase platelets. Figure 5b shows a close-up image of the magnetization texture around intersections between the three phases for the region marked with a square in Fig. 5a. Via fitting a magnetization profile across the DW by $\tanh \left( 2r/\delta _ {\rm DW} \right)$, where *r* is the distance from the DW center^31^, we deduced the DW-width parameter in the $\hbox {SmCo}_5$ and the $\hbox {Sm}_2\hbox {Co}_{{17}}$ phases to be 1.5 nm and 4.7 nm, respectively. These values are slightly larger than the theoretically expected values of 1.2 nm and 2.7 nm, but they agree with our experimental observation of $4 \pm 2$ nm (see Supplementary Figure 2). This confirms our conclusion that the DWs are mostly located in $\hbox {Sm}_2\hbox {Co}_{{17}}$ and pinned to the $\hbox {SmCo}_5$ cell walls. The minimum domain size of 5 nm is also shown in Fig. 5b at the left edge of the $\hbox {SmCo}_5$ cell wall, which is intersected by a Z-phase platelet. Notably, the DWs inside the Z phase are extremely thin, and the moments turn away from the *c*-axis due to the dominating shape anisotropy of the platelets. This indicates that the DWs between the Sm–Co phases and the Z phase are in fact $\pi$/2 DWs. To minimize the associated exchange energy, the magnetic moments are twisted with opposite handedness at the edges of the $\hbox {SmCo}_5$ cell-wall boundary.

This is further analyzed in Fig. 5c, which shows a detailed view of a region where the three phases intersect and a DW propagates through all of them. Here we observe a narrow DW in the $\hbox {SmCo}_5$ cell wall, a broader DW in the $\hbox {Sm}_2\hbox {Co}_{{17}}$ matrix, and a curling of the moments away from the *c*-axis inside the Z phase. The curling has a significant out-of-plane component, and the DW is injected into the hard phase at a location where the three phases meet. These results shed new light on previous observations based on electron microscopy^23^, which suggested an out-of-plane tilting of the magnetic flux away from the easy axis around the various intersections.

The points in the microstructure where the three phases intersect play a critical role in the magnetization process because their edges, having different material properties, enable curling instabilities, where DWs can be injected into or ejected out of the material. We show in Fig. 5d–g the process of magnetization reversal, coming from a saturated state and applying an external field in the opposite direction. The demagnetization starts at the intersections between the Z phase and the $\hbox {Sm}_2\hbox {Co}_{{17}}$ matrix in the form of nucleating domains, which gradually grow inside the $\hbox {Sm}_2\hbox {Co}_{{17}}$ matrix and become pinned by the hard $\hbox {SmCo}_{{5}}$ cell walls. The domain growth then progresses through the intersections where all three phases meet and crosses the $\hbox {SmCo}_{{5}}$ cell walls through these points. This further indicates that the Z phase does indeed play a vital role in the magnetization process, which is an interplay between curling instabilities at the intersections between the Z phase and the $\hbox {Sm}_2\hbox {Co}_{{17}}$ matrix and pinning at the cell walls. Importantly, this demagnetization process might also be responsible for the formation of DWs with higher winding angles, such as those observed in Fig. 2.

## Discussion

We have shown via magnetic imaging in TEM, as well as APT and micromagnetic simulations, that there are sharp magnetic DWs in cellular Sm–Co magnets, which follow exactly the morphology of the $\hbox {SmCo}_5$ cell walls and are offset by the Z-phase platelets. Our findings indicate that the DWs are mostly located in the $\hbox {Sm}_2\hbox {Co}_{{17}}$ matrix and that the coercivity does not change with the $\hbox {SmCo}_{{5}}$ cell-wall thickness as long as (1) the thickness is larger than the DW width and (2) the distribution of elements (especially Cu) in the cell walls does not vary with the thickness. Based on this, we propose that the cell-wall thickness be reduced to a minimum in order to increase the remanence, given that $\hbox {Sm}_2\hbox {Co}_{{17}}$ has higher remanence than $\hbox {SmCo}_5$. Furthermore, we have revealed that curling instabilities at the intersections between the matrix and the Z phase act as nucleation sites for DWs upon switching the external magnetic field. After nucleation, the DWs propagate inside the $\hbox {Sm}_2\hbox {Co}_{{17}}$ matrix and become pinned at the $\hbox {SmCo}_{{5}}$ cell walls. They can only propagate further through intersections between the cell walls and the Z phase. Therefore, the Z phase plays a key role in initiating the magnetization reversal. Since Zr is essential in forming the Sm–Co microstructure, it cannot be completely eliminated from the material, but the Z-phase platelets should be as thin as possible to increase the coercivity of the material. With an appropriate modification of the chemical composition, the heat-treatment process may also have to be refined to achieve this desired microstructure. Finally, we have observed topologically non-trivial domains with highly complex DWs, and, where all three phases meet, out-of-plane curling of DWs. These exotic magnetic structures should be studied further to understand the physics of multi-phase magnets and to harness the full potential of these high-performance materials.

## Methods

### Sample synthesis

For the synthesis of the Sm–Co magnet, the alloying elements were melted in an induction furnace under argon atmosphere (99.999% purity), and the resulting alloy was cast in a metallic mold. After crushing the alloy with a hammer mill, the resulting powder was processed in a jet mill towards a particle size of 4–8 $\upmu$m. The powder was then filled into a rubber mold, aligned with magnetic pulses of field strength 5 T and then pressed in an isostatic press at 300 MPa. The green parts were sintered under vacuum at a temperature of 1200–1220 $^\circ$C, solution annealed at 1170–1200 $^\circ$C, and then quenched with an inert gas to room temperature. Subsequently, the parts were tempered at 850 $^\circ$C, slowly cooled to 400 $^\circ$C, and quenched to room temperature. The material was produced by Arnold Magnetic Technologies, and has an overall chemical composition of Sm($\hbox {Co}_{0.695}\hbox {Fe}_{0.213}\hbox {Cu}_{0.070}\hbox {Zr}_{0.022}$)$_{7.570}$ with a minor additional oxygen content in the form of $\hbox {Sm}_2\hbox {O}_3$.

### Magnetometry

The magnetization of a small sample piece was measured along the easy axis as a function of the external field at room temperature using a Superconducting Quantum Interference Device (SQUID) in a Magnetic Property Measurement System (MPMS3) of Quantum Design.

### Transmission electron microscopy

Electron-transparent specimens for TEM studies were prepared using $\hbox {Ga}^+$ sputtering and a conventional lift-out method in a Helios 600i dual-beam focused ion beam (FIB) scanning electron microscope (SEM) workstation. The ion-beam induced damage on the surfaces was reduced by low-energy (< 1 keV) $\hbox {Ar}^+$ milling using a Fischione Nanomill system. The thickness of the lamellae was measured on an FEI Tecnai F30 FEG transmission electron microscope using an electron energy-loss spectroscopy (EELS) log-ratio technique. A uniformly varying range of thicknesses between 80 and 140 nm was achieved.

The Sm–Co specimens were studied at remanence in magnetic-field-free (Lorentz mode) conditions using a spherical aberration-corrected FEI Titan microscope operated at 300 kV. In Fresnel-mode LTEM images, the intensity distribution at defocus $\delta z$ is recorded to reveal a bright (convergent) or dark (divergent) contrast at the positions of the magnetic DWs. The net deflection of electrons from the magnetic domains is induced by the Lorentz force, $\mathbf{F} = -e\mathbf{v} \times \mathbf{B}$, where *e* is the electron charge, $\mathbf{v}$ is the velocity vector of the incident electrons and $\mathbf{B}$ is the in-plane magnetic induction in the sample. A conventional microscope objective lens was used to apply a magnetic field on the specimen. TEM images were recorded using a direct-electron counting Gatan K2-IS camera and Gatan Microscopy Suite software. Electron holograms were recorded in Lorentz mode using a biprism positioned in one of the conjugated image planes of the electron column. The biprism voltage used was typically in the range of 90–100 V, which forms a fringe spacing of 3 nm with a contrast of 75%.

### Micromagnetic simulations

High-resolution micromagnetic simulations were performed to investigate the link between the microstructure and the domain-wall network in Sm–Co magnets. The total energy density of the system consists of (1) ferromagnetic exchange; (2) uniaxial magnetocrystalline anisotropy; (3) Zeeman coupling to an external magnetic field; (4) dipole-dipole interactions; and (5) ferromagnetic exchange between different phases:

1 $$\begin{aligned} F = \sum _i \left[ A^i ({{\nabla \mathbf{m}}^i})^2-K_\text {u}^i(m_c^i)^2 + \mu _0 M_\text {s}^i{\mathbf{m}^i \cdot \mathbf{H}}_{\text {ext}}- \frac{\mu _0 M_ {\rm s}^i}{2}{\mathbf{m}^i \cdot \mathbf{H}}_{\text {dem}}^i - \mu _0 M_\text {s}^i{\mathbf{m}^i \cdot \mathbf{H}}_{\text {exc}} \right] , \end{aligned}$$

where $\mathbf{m}^i=\mathbf{M}^i/M_\text {s}^i$ is the magnetization unit vector of phase *i* with $M_\text {s}^i$ the saturation magnetization, $A^i$ is the exchange stiffness, $K_\text {u}^i$ is the first-order uniaxial anisotropy constant, $\mathbf {H}_{\text {ext}}$ is the external magnetic field, $\mathbf {H}_{\text {dem}}$ is the local demagnetizing field due to dipole-dipole interactions, and $\mathbf{H}_{\text {exc}}$ describes the exchange between the different phases. The *c*-component of the magnetization is inside the lamella plane.

The material parameters were taken from the literature^6,25,32^, and are $\mu _0 M_ {\rm s} = 1.05$ T, $A = 23.6$ pJ $\hbox {m}^{-1}$, and $K_ {\rm u} = 17.2$ MJ $\hbox {m}^{-3}$ for $\hbox {SmCo}_5$; $\mu _0 M_ {\rm s} = 1.25$ T, $A = 24.7$ pJ $\hbox {m}^{-1}$, and $K_ {\rm u} = 3.3$ MJ $\hbox {m}^{-3}$ for $\hbox {Sm}_2\hbox {Co}_{{17}}$; and $\mu _0 M_ {\rm s} = 0.37$ T, $A = 11$ pJ $\hbox {m}^{-1}$, and $K_ {\rm u}$(Z phase)$<< K_ {\rm u}$($\hbox {Sm}_2\hbox {Co}_{{17}}$ or $\hbox {SmCo}_5$). Note that the element Cu peaks at 43 at% in the $\hbox {SmCo}_5$ cell walls, effectively lowering their $M_ {\rm s}$, *A* and $K_ {\rm u}$^24^. However, we do not expect this to impact our results, because Cu segregates at the cell walls in a Gaussian-like manner, meaning that the Cu concentration at the cell-wall edges is low. If the DWs are located in the $\hbox {Sm}_2\hbox {Co}_{{17}}$ matrix and pinned at the cell walls, only the magnetic properties of the cell-wall edges are expected to determine the strength of the DW pinning. We have successfully validated this assumption by performing a series of simulations in which the values of *A* and $K_ {\rm u}$ were reduced for the cell walls. As shown in Supplementary Figure 3, the micromagnetic simulations only match the experimental results well if the magnetic properties of the cell walls are close to those of pure $\hbox {SmCo}_5$. The simulations were also tested for lamellae thicknesses between 50 and 100 nm, and qualitatively the same results were obtained.

The exchange field between two phases *i* and *j* is proportional to $\frac{A^i}{M_ {\rm s}^i}\frac{A^j}{M_ {\rm s}^j}/\left( {\frac{A^i}{M_ {\rm s}^i}+\frac{A^j}{M_ {\rm s}^j}}\right)$. Based on our optimization, described in the main text and shown in Fig. 3e, we found the following exchange values: (1) cell walls to matrix: 16 pJ $\hbox {m}^{-1}$; (2) matrix to cell walls: 13 pJ $\hbox {m}^{-1}$; (3) matrix to Z phase: 4.4 pJ $\hbox {m}^{-1}$; (4) Z phase to matrix: 1.3 pJ $\hbox {m}^{-1}$; (5) cell walls to Z phase: 2.7pJ $\hbox {m}^{-1}$; and (6) Z phase to cell walls: 0.95 pJ $\hbox {m}^{-1}$.

Using Eq. (1), we solved the Landau–Lifshitz–Gilbert (LLG) equation of motion

2 $$\begin{aligned} \partial _{t} \mathbf{m}=-\gamma (\mathbf{m} \times \mathbf{H}_{\text {eff}})+\alpha (\mathbf{m} \times \partial _{t} \mathbf{m}) \;, \end{aligned}$$

where $\gamma$ is the electron gyromagnetic ratio, $\mathbf{H}_{\text {eff}}=-\partial _{\mathbf{m}} F / \mu _{0} M_\text {S}$ is the effective magnetic field in the material, consisting of external and internal magnetic fields, and $\alpha$ is the dimensionless damping parameter. The simulations were done with mumax3^38^, and the visualization of the magnetization textures was done with Paraview^39^. The computational cell was generally 1 nm, but it was varied between 0.8 and 2 nm to verify the numerical stability of the system.

### Atom probe tomography

The needle-shaped geometry required for APT analysis was prepared by applying standard lift-out practices using an FEI Helios Focused Ion Beam 600i workstation, and mounting the needle onto a flat-top microtip coupon supplied by Cameca. Sequential annular milling was applied to achieve an apex of < 70 nm diameter, including low-kV cleaning, resulting in < 0.01 at% Ga in the top 10 nm of the specimen. Data collection was performed using a LEAP4000X-HR instrument applying 100 pJ laser-pulse energy with 200 kHz repetition rate and a specimen temperature of 54 K, resulting in a $\hbox {Co}^{++}$/$\hbox {Co}^+$ charge-state ratio between 5 and 10. With these parameters and a chamber vacuum level of 10^−9^ Pa, data were collected between 5 and 9.5 kV with a background level consistently below 20 ppm $\hbox {ns}^{-1}$. The atom-map reconstruction was validated by considering that the Z-phase platelets are atomically flat, and spatial distribution maps were performed along the *c*-axis (normal to the platelets) to measure the lattice spacings and thereby to validate the accuracy of the atom-map reconstruction dimensions.

### Magnetic phase image and LTEM simulations

The electromagnetic phase shift induced in an electron wave by passing through a sample is described by the Aharonov–Bohm effect and can be expressed as^40^:

3 $$\begin{aligned} \varphi (x,y) =\varphi _{ {\rm el}}(x,y)+\varphi _{ {\rm mag}}(x,y) = C_{ {\rm el}}\int V(\mathbf{r})dz-\frac{\pi }{\Phi _{0}}\int A_{z}(\mathbf{r})dz, \end{aligned}$$

with $\varphi _{ {\rm el}}\left( x,y\right)$ and $\varphi _{ {\rm mag}}\left( x,y\right)$ denoting the electrostatic and magnetic contributions to the phase shift, the interaction constant $C_{ {\rm el}}=\frac{\gamma m_{ {\rm el}}e\lambda }{\hbar ^{2}}$, the magnetic flux quantum $\Phi _{0}=\pi \hbar /e$, the Lorentz factor $\gamma$, the electron rest mass $m_{ {\rm el}}$ and the electron wavelength $\lambda$. Furthermore, $A_{z}\left( \mathbf{r}\right)$ with $\mathbf{r}=\left( x,y,z\right)$ is the *z* component of the magnetic vector potential $\mathbf{A}({\mathbf{r}})$, where *z* corresponds to the incident electron beam direction^41,42^.

The magnetization $\mathbf{M}(\mathbf{r})$ in the sample is linked to the vector potential by the vector convolution integral^43^

4 $$\begin{aligned} \mathbf{A}(\mathbf{r}) =\frac{\mu _{0}}{4\pi }\int \mathbf{M}(\mathbf{r'})\times \frac{\mathbf{r}-\mathbf{r}'}{|\mathbf{r}-\mathbf{r}'|^{3}}d\mathbf{r}', \end{aligned}$$

where $\mu _{0}$ is the vacuum permeability. Using both equations, the magnetic phase shift can be expressed in terms of the magnetization as:

5 $$\begin{aligned} \varphi _{ {\rm mag}}\left( x,y\right) =-\frac{\mu _{0}}{2\Phi _{0}}\int \frac{\left( y-y'\right) M_{x}(\mathbf{r}')-\left( x-x'\right) M_{y}(\mathbf{r}')}{\left( x-x'\right) ^{2}+\left( y-y'\right) ^{2}}d\mathbf{r}'. \end{aligned}$$

By discretizing this equation and utilizing known analytical solutions for the magnetic phase of simple magnetized geometries, magnetic phase images $\varphi _{ {\rm mag}}\left( x,y\right)$ can be calculated for arbitrary magnetization distributions $\mathbf{M}(\mathbf{r})$^44^.

“Contour maps” are used for the visualization of the magnetic phase in the form of magnetic induction. They are generated in Figs. 2 and 4 by taking the cosine of the magnetic phase $\varphi _{ {\rm mag}}$, which can be amplified beforehand to increase the number of fringes for visualization purposes. A color scheme is superimposed on the magnetic induction maps, which is determined by the gradient of $\varphi _{ {\rm mag}}$. The latter is an indicator of the direction of the projected in-plane magnetic induction and is shown in Figs. 2e and 4d as a color wheel. The phase difference between two neighboring contours is 2$\pi$.

The magnetic phase $\varphi _{ {\rm mag}}$ can further be utilized to simulate LTEM images by convoluting the corresponding wave function $\Psi \left( x,y\right) =e^{i\varphi _{ {\rm mag}}\left( x,y\right) }$ with a phase plate:

6 $$\begin{aligned} \Psi _{ {\rm LTEM}}\left( x,y\right) =\mathscr {F}_{2}^{-1}\left\{ \mathscr {F}_{2}\left\{ e^{i\varphi _{ {\rm mag}}\left( x,y\right) }\right\} \cdot e^{-i\chi \left( q_{x},q_{y}\right) }\right\} , \end{aligned}$$

with $\mathscr {F}_{2}\left\{ ...\right\}$ denoting the 2D Fourier transform, $\mathscr {F}_{2}^{-1}\left\{ ...\right\}$ its inverse, and $\chi \left( q_{x},q_{y}\right)$ denoting an aberration function^45^ in the diffraction space containing the defocus $C_{1}$ (with positive $C_{1}$ referring to overfocus) given by:

7 $$\begin{aligned} \chi \left( q_{x},q_{y}\right) =\pi \lambda C_{1}\left( q_{x}^{2}+q_{y}^{2}\right) . \end{aligned}$$

The LTEM images are then calculated from the corresponding electron wave by:

8 $$\begin{aligned} I_{ {\rm LTEM}}\left( x,y\right) =\Psi _{ {\rm LTEM}}\left( x,y\right) \cdot \Psi _{ {\rm LTEM}}^{*}\left( x,y\right) . \end{aligned}$$

## Supplementary information

Supplementary material 1

Supplementary material 2
