# Supplementary material for: Unconventional magnetization textures and domain-wall pinning in Sm–Co magnets
Source: Sci Rep. 2020 Dec 3;10:21209. doi: 10.1038/s41598-020-78010-0 (PMC7713442; doi:10.1038/s41598-020-78010-0)
Supplement: Supplementary file 1 — Supplementary material 1 [file 41598_2020_78010_MOESM1_ESM.pdf]

Unconventional magnetization textures and domain-wall pinning in Sm–Co magnets

Leonardo Pierobon[1,*], András Kovács[2], Robin E. Schäublin[1,3], Stephan S. A. Gerstl[1,3], Jan Caron[2], Urs V. Wyss[4], Rafal E. Dunin-Borkowski[2], Jörg F. Löffler[1,*], and Michalis Charilaou[1,5,*]

[1]Laboratory of Metal Physics and Technology, Department of Materials, ETH Zurich, Zurich, 8093, Switzerland
[2]Ernst Ruska-Centre for Microscopy and Spectroscopy with Electrons, and Peter Grünberg Institute, Forschungszentrum Jülich, 52425 Jülich, Germany
[3]Scientific Center for Optical and Electron Microscopy, ETH Zurich, 8093 Zurich, Switzerland
[4]Arnold Magnetic Technologies, 5242 Birr-Lupfig, Switzerland
[5]Department of Physics, University of Louisiana at Lafayette, Lafayette, LA 70504, USA
[*]Leonardo Pierobon, leonardo.pierobon@mat.ethz.ch; Jörg F. Löffler, joerg.loeffler@mat.ethz.ch; Michalis Charilaou, michalis.charilaou@louisiana.edu

Diffraction analysis

Supplementary Figure 1 shows, for the sample in Figure 1 of the main text, a diffraction pattern that contains reflections from [110] and [$\bar{1}\bar{1}$0] directions of Sm_2Co_{17}, proving the existence of twinning. The experimental and simulated patterns reveal an excellent agreement.

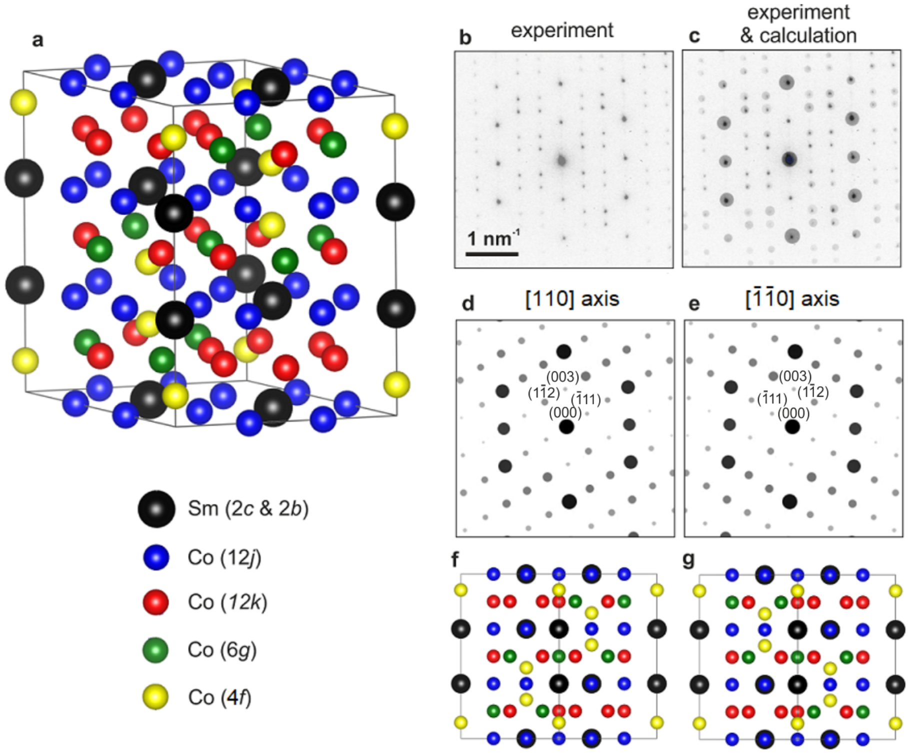

Supplementary Figure 1. Crystal structure of Sm–Co. (a) Unit cell of the Sm_2Co_{17} phase showing the atomic sites 2b and 2c (black) for Sm, and 12j (blue), 12k (red), 6g (green) and 4f (yellow) for Co. (b) Recorded diffraction pattern from one Sm_2Co_{17} cell and (c) calculated pattern overlaid on the experimental pattern, illustrating excellent agreement. The calculated pattern is a result of twinning between (d) [110] and (e) [$\bar{1}\bar{1}$0] directions, and the corresponding crystal structure viewed along these directions is shown in (f) and (g), respectively.

Domain-wall width

The full width at half maximum (FWHM) of the domain-wall (DW) contrast of 4 ± 2 nm at zero defocus was extrapolated from a series of LTEM images recorded at different defocus values, as shown in Supplementary Figure 2. This approach may give a slight overestimate of the true width.

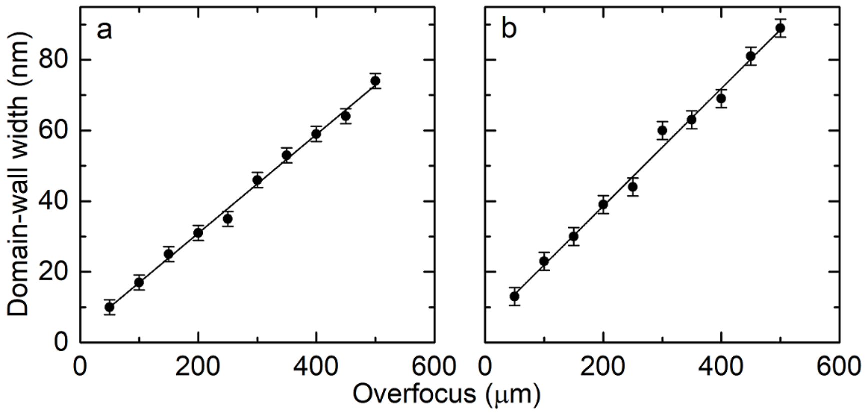

Supplementary Figure 2. Measurements of DW width. (a, b) The FWHM of two different DWs was obtained from a focal-series reconstruction. The apparent width of divergent DWs (appearing as dark contrast) was measured for different values of defocus (data points). The real width was then deduced from the intersection of the linear fit to the data points with the *y*-axis.

The effect of a Cu gradient on the magnetic properties of $SmCo_5$ cell walls

As shown in Figure 1i of the main text, Cu segregates inside the $SmCo_5$ cell walls in a Gaussian-like manner and peaks at 43 at.%. The Cu content significantly lowers the values of the saturation magnetization, exchange stiffness A and magnetocrystalline anisotropy K_u of the cell walls. However, the Cu concentration is low at the cell-wall edges, so that the magnetic properties of the edges are close to those of $SmCo_5$. If the DWs are located in the Sm_2Co_{17} phase and pinned at the cell walls, then only the magnetic properties of the cell-wall edges are expected to impact the strength of the DW pinning and are therefore considered in the simulations. In order to validate this assumption, we performed a series of simulations where we reduced the values of A and K_u of the cell walls.

We consider the cases: (1) K_u(cell walls) > K_u(Sm_2Co_{17}) and (2) K_u(cell walls) < K_u(Sm_2Co_{17}). Supplementary Figure 3 shows an example for each case, where the values for A and K_u of the cell walls are 50% (Case 1) and 10% (Case 2) of those given for $SmCo_5$ in the Methods section of the main text. As can be seen in Supplementary Figure 3, the simulated hysteresis loops do not reproduce well the experimental results. Specifically, the coercivity is lower for case 1 and higher for case 2, and the characteristic dip in the magnetization before its reversal is too small. For case 1, a DW pattern similar to that observed experimentally can be seen, but the deviations from the experiment are much larger than for the sample without reduction (Supplementary Fig. 3b,c). For case 2, the DW pattern cannot be replicated at all, which renders case 2 physically incorrect. These results confirm our assumption that the magnetic properties of the cell-wall edges determine the strength of the DW pinning. A finer micromagnetic model, in which the magnetic properties in the interior of the cell walls would be reduced, may be realized, but this would have no relevance for the findings in this paper, because it is the magnetic properties of the cell-wall edges that affect the DW-pinning strength.

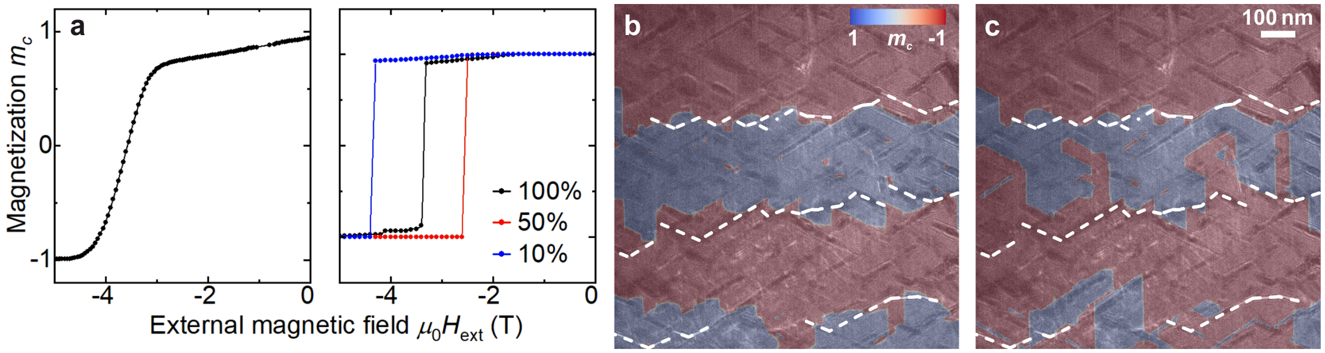

Supplementary Figure 3. The effect of Cu on the magnetic properties. (a) Comparison of experimental (left panel) and simulated (right panel) hysteresis loops with the cell-wall magnetic properties being 100%, 50% and 10% of those of $SmCo_5$, revealing that the experiments and theory best match for 100%. An LTEM image with the positions of the DWs marked white is superimposed on the simulated magnetic structure for (b) 100% (identical to Figure 4b in the main text) and (c) 50%. The deviation from the experiment is significantly larger for 50%. For 10%, the DW pattern cannot be replicated at all in the simulations.

Atom-probe tomography reconstruction

An atom-probe tomography (APT) reconstruction is presented as Supplementary Video 1. Isoconcentration surfaces of 9.83 at.% Zr indicate flat Z-phase platelets, while the isoconcentration surfaces of 14.51 at.% Sm reveal a twisted $SmCo_5$ cell wall. The twisting of the cell wall projected in two dimensions results in both blurry and sharp contrast, as observed by transmission electron microscopy (Figure 1a of the main text). The reconstruction also reveals that Cu accumulates inside the cell walls (illustrated in pink).

Supplementary Video 1. APT reconstruction of Sm–Co. Atomic positions of Co (green), Fe (blue), Sm (red), Cu (pink) and Zr (orange) are plotted together with the isoconcentration surfaces of 9.83 at.% Zr and 14.51 at.% Sm. The reconstruction reveals flat Zr-rich Z-phase platelets, a twisted $SmCo_5$ cell wall, and an accumulation of Cu inside the cell wall.
